# Dose-Escalated Hypofractionated Intensity-Modulated Radiotherapy in High-Risk Carcinoma of the Prostate: Outcome and Late Toxicity

**DOI:** 10.1155/2012/450246

**Published:** 2012-06-20

**Authors:** David Thomson, Sophie Merrick, Ric Swindell, Joanna Coote, Kay Kelly, Julie Stratford, James Wylie, Richard Cowan, Tony Elliott, John Logue, Ananya Choudhury, Jacqueline Livsey

**Affiliations:** ^1^Department of Clinical Oncology, The Christie NHS Foundation Trust, Manchester M20 4BX, UK; ^2^University of Manchester, Manchester M13 9PL, UK; ^3^Department of Clinical Oncology, Raigmore Hospital, Inverness IV2 3UJ, UK

## Abstract

*Background*. The benefit of dose-escalated hypofractionated radiotherapy using intensity-modulated radiotherapy (IMRT) in prostate cancer is not established. We report 5-year outcome and long-term toxicity data within a phase II clinical trial. *Materials and Methods*. 60 men with predominantly high-risk prostate cancer were treated. All patients received neoadjuvant hormone therapy, completing up to 6 months in total. Thirty patients were treated with 57 Gy in 19 fractions and 30 patients with 60 Gy in 20 fractions. Acute and 2-year toxicities were reported and patients followed longitudinally to assess 5 year outcomes and long-term toxicity. Toxicity was measured using RTOG criteria and LENT/SOMA questionnaire. *Results*. Median followup was 84 months. Five-year overall survival (OS) was 83% and biochemical progression-free survival (bPFS) was 50% for 57 Gy. Five-year OS was 75% and bPFS 58% for 60 Gy. At 7 years, toxicity by RTOG criteria was acceptable with no grade 3 or above toxicity. Compared with baseline, there was no significant change in urinary symptoms at 2 or 7 years. Bowel symptoms were stable between 2 and 7 years. All patients continued to have significant sexual dysfunction. *Conclusion*. In high-risk prostate cancer, dose-escalated hypofractionated radiotherapy using IMRT results in encouraging outcomes and acceptable late toxicity.

## 1. Introduction

Dose-escalated radiotherapy improves local and biochemical disease control in localised prostate cancer [1–4]. However, this is at the expense of increased late normal tissue toxicity and overall treatment time [3–6].There is increasing evidence that the *α*/*β* ratio for prostate cancer may be low [7–9], and in one analysis of nearly 6000 patients the calculated *α*/*β* ratio was 1.4 [10]. This suggests that a hypofractionated regimen should be biologically advantageous. A shortened overall treatment time also provides benefits in terms of patient acceptability and health economics [11].

Our group has previously published data on patients treated with 50 Gy in 16 daily fractions (equivalent total dose of 66 Gy, assuming an *α*/*β* ratio for prostate cancer of 1.5) [12]. However, the biochemical outcome for patients with intermediate or high risk disease was inferior to dose-escalated series using 2 Gy per fraction [13]. This finding was replicated in a later study using low-dose hypofractionated radiotherapy [14]. Although there is evidence for improved bPFS with increasing doses of radiotherapy, no overall survival benefit has yet been demonstrated. Indeed, the MRC RT01 study showed equivalent overall survival at 10 years between 64 Gy in 32 fractions and 74 Gy in 37 fractions despite a continued significant improvement in biochemical-free progression [15]. However, the evidence for a dose-effect above 70 Gy using conventional fractionation with a resultant increase in overall treatment time has led to interest in dose-escalated hypofractionated radiotherapy. Despite this, published toxicity and survival outcomes are limited [16–18].

One strategy to overcome potential toxicity is the use of intensity-modulated radiotherapy (IMRT), which reduces the volume of normal tissue irradiated by improving the dose distribution and conformity of delivered radiation [19, 20]. We have previously published results demonstrating that dose-escalated hypofractionated radiotherapy using IMRT in treatment of predominantly high-risk prostate cancer is well tolerated with minimal acute side effects and acceptable toxicity 2 years following treatment [21]. Here we report our 5-year outcome and 7-year late toxicity data, assessed using a validated patient questionnaire [22], in patients treated within this early phase clinical trial.

## 2. Materials and Methods

### 2.1. Patients

Men with World Health Organisation performance status 0-1, histologically confirmed prostate cancer who were stage T3N0M0 (using pelvic magnetic resonance imaging and technetium 99 bone scintigraphy as staging investigation) with any Gleason score and prostate-specific antigen (PSA) ≤50 *μ*g/L or T2N0M0 with Gleason score ≥7 and/or PSA 20–50 *μ*g/L were eligible. The 1997 American Joint Committee on Cancer (AJCC) staging system was used. Patients were followed up longitudinally on a 6 monthly basis to assess long-term toxicity and outcome, measured by biochemical progression-free survival (bPFS) and cause-specific and overall survival.

### 2.2. Treatment

Sixty men were recruited and treated from May 2002 to September 2003. All patients received neoadjuvant hormone therapy with either goserelin acetate 3.6 mg subcutaneously every 28 days or bicalutamide 150 mg daily for 3 months before radiotherapy, completing up to a total of 6 months. The first 30 patients were entered in to dose level one and received 57 Gy in 19 daily fractions over 4 weeks and the second cohort of 30 patients was treated with 60 Gy in 20 daily fractions over 4 weeks.

Patients were treated supine, with an empty bladder and without formal immobilisation; the radiotherapy CT planning scan was performed in the treatment position from the L5-S1 interface to 10 cm caudal to ischial tuberosities with a slice thickness of 5 mm. Clinical Target Volume (CTV1) encompassed the prostate and seminal vesicles and CTV2 the prostate alone. The outer rectal wall was contoured from the rectosigmoid junction to the anorectal junction and outer bladder wall contoured in its entirety. A planning target volume (PTV) was generated by addition of a 1 cm margin to CTV1 except at the prostate-rectum interface where the margin was 0.7 cm.

IMRT was inverse-planned as previously described [23], using five isocentric fields with posterior, right lateral oblique, right anterior oblique, left anterior oblique, left lateral oblique fields (180°, 260°, 325°, 35°, 100° fields, resp.). Dose was prescribed to the mean PTV. IMRT was delivered using an 8 MV step-and-shoot multileaf collimator once daily, 5 days per week. Treatment verification was performed using cone-beam scans on days 1 to 3 and weekly thereafter, unless otherwise clinically indicated. Dose parameters for target volumes and organs at risk have been previously reported [21].

### 2.3. Outcome and Toxicity

Outcomes were assessed at 5 years from the completion date of radiotherapy. bPFS, defined by the Phoenix criteria (failure at nadir PSA +2 *μ*g/L) [24], cause-specific survival, and overall survival for the two radiotherapy schedules were reported.

Toxicity was assessed at median followup of 7 years (84 months; range, 13–93 months) from the date of radiotherapy. Outcomes derived from the Radiation Therapy Oncology Group (RTOG) objective system for bowel and urinary function were collected by retrospective review of case-notes, completed by a physician or specialist nurse in outpatient clinic. Results were presented as maximum scores recorded for each category. Prospective assessment of bowel, bladder, and sexual function toxicity was performed using a validated late effects in normal tissues subjective, objective, management, and analytic scales (LENT/SOMA; subjective part) questionnaire, where toxicity is reported on a 4-point scale (a score of 0 represents no toxicity and a score ≥2 denotes significant toxicity) [22]. Questionnaires were completed by patients and returned by post. Median and maximum scores for each symptom category using data returned from all patients were presented and compared with results collected from the same patients at 2-year followup and baseline (defined as at the start of radiotherapy treatment).

### 2.4. Statistical Analysis

Log rank (Mantel-Cox) analysis was used to compare bPFS, cause-specific survival and overall survival for the two radiotherapy schedules and illustrated using Kaplan-Meier plots. Date of death was used or observations censored at the last date that the patient was seen. A univariate Cox regression analysis was performed on all patients to identify prognostic factors for disease outcome.

LENT/SOMA average and maximum scores at 7-year followup, 2 years follow-up and baseline were compared using Friedman two-way analysis of variance by rank. Where there was significant variation, further comparisons between results at baseline, and 2 years, 2 years and 7 years and baseline and 7 years of followup were performed using Wilcoxon matched pairs signed rank test using a Bonferroni correction (*α*/3; *α* = 0.05, new *α* = 0.017). LENT/SOMA maximum scores for each symptom subscale were divided into nonsignificant symptoms (scores of 0, 1) and significant symptoms (scores of ≥2) and results for each patient compared between baseline and 2 years, 2 years and 7 years and baseline and 7 years of followup using three McNemar's tests, each with the above Bonferroni correction.

## 3. Role of the Funding Source

The sponsor had no role in the study design; collection, analysis, or interpretation of data; writing the report; or decision to submit the paper for publication.

## 4. Results

### 4.1. Patient Characteristics

Baseline patient characteristics are presented in Table 1. Mean age at enrolment was 68 years (range, 57–80 years) and median pretreatment PSA was 20.4 *μ*g/L (range, 3.5–58.0 *μ*g/L). Patients were retrospectively assigned to prognostic groups according to the D'Amico classification system and 59 (98%) patients had high risk and 1 (2%) patient intermediate risk prostate cancer [25].

### 4.2. Outcome

**Figure 1:**
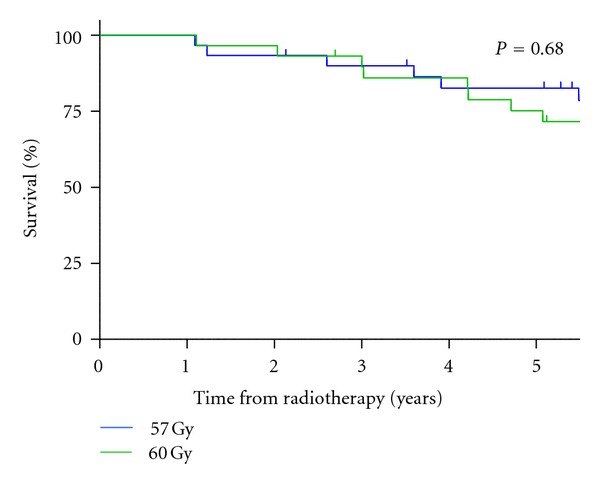
Kaplan-Meier estimates for overall survival for the two radiotherapy fractionation schedules, 57 Gy in 19 fractions and 60 Gy in 20 fractions.

**Figure 2:**
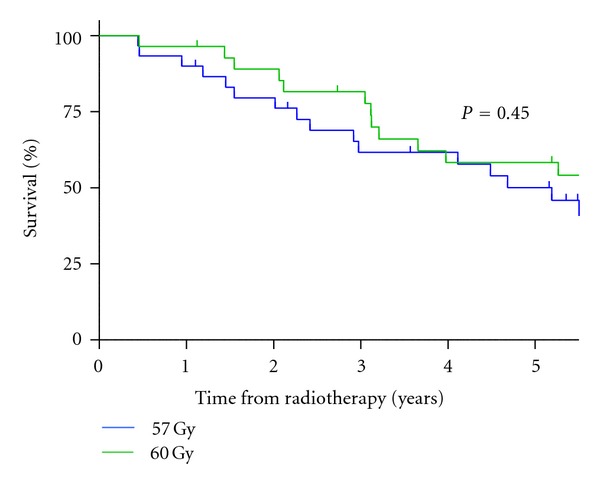
Kaplan-Meier estimates for biochemical progression-free survival for the two fractionation schedules, 57 Gy in 19 fractions and 60 Gy in 20 fractions.

Acute toxicity and toxicity at two years in patients treated within this trial have previously been published [21]. Forty-four of sixty patients were alive at a median followup of 7 years (84 months; range, 13–93 months). Eleven (18%) died from prostate cancer and 5 (8%) from an intercurrent disease, all of whom were in the 60 Gy in 20 daily fractions group. No patients were lost to followup. For those treated with 57 Gy in 19 daily fractions, overall survival at 5 years was 82.7% (95% confidence interval (CI): 63.1–92.5; Figure 1), cause-specific survival 82.7% (95% CI: 63.1–92.5), and bPFS 50.1% (95% CI: 30.6–66.8; Figure 2). In men treated with 60 Gy in 20 fractions, overall survival at 5 years was 75.2% (95% CI: 55.0–87.3; Figure 1), cause-specific survival 84.1% (95% CI: 63.0–93.7), and bPFS 58.3% (95% CI: 37.5–74.3; Figure 2). There was no significant difference in overall survival, cause-specific survival or bPFS between the fractionation schedules. None of the known prognostic factors (presenting PSA, Gleason score, T stage, or patient age) was significantly associated with improvement in cause-specific survival.

### 4.3. Toxicity

Nine (21%) patients experienced RTOG grade 1 bowel or bladder toxicity and there was no grade 2 toxicity or above. There was no difference between the fractionation schedules. LENT/SOMA questionnaires were returned and available for analysis for 28/44 patients, 14/28 for each fractionation schedule. Of those who returned questionnaires, some men chose not to answer certain questions, particularly those relating to sexual function.

**Figure 3:**
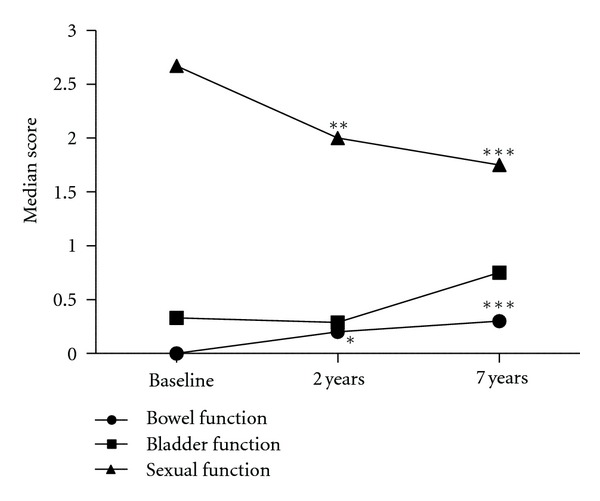
Late effects in normal tissues subjective, objective, management, and analytic scales (LENT/SOMA) questionnaires data. Line graph showing median scores at baseline, 2-year and 7-year followup (bowel function, *baseline to 2 years *P* = 0.015, ***baseline to 7 years, *P* < 0.001; sexual function, **baseline to 2 years *P* < 0.01; **2 years to 7 years *P* < 0.01 (not shown), ***baseline to 7 years *P* < 0.001).

**Figure 4:**
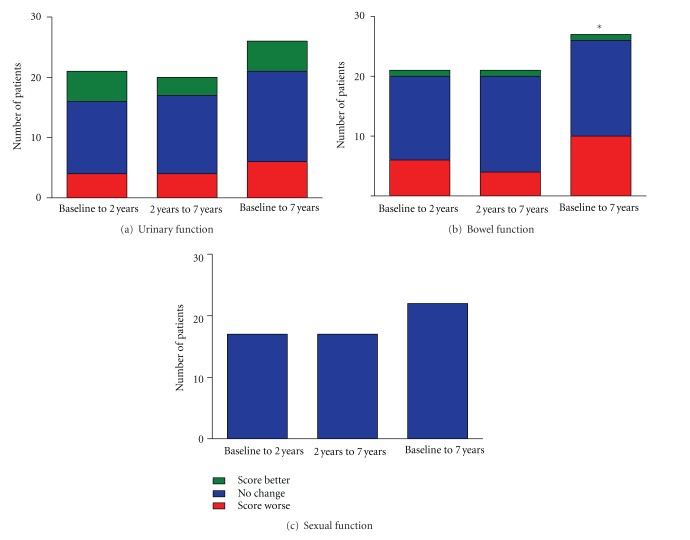
Late effects in normal tissues subjective, objective, management and analytic scales (LENT/SOMA) data. Change in maximum scores per symptom area between nonsignificant scores (score 0,1) and significant scores (≥2) at baseline, 2-year and 7-year followup (Figure 4(b). Bowel function, *baseline to 7 years *P* = 0.012).

For urinary symptoms, LENT/SOMA median scores were less than 1 at baseline, 2 years, and 7 years (Figure 3). There was an increasing trend in median score but this was not significant (*P* = 0.175). There was a high degree of baseline urinary dysfunction in this cohort (19/27 patients recorded a maximum score ≥2 at baseline) and there was no significant change in maximum urinary symptom scores at 2 years or 7 years (*P* = 0.125; Table 2). However, at 7 years one patient treated with 60 Gy in 20 daily fractions did report significant urinary symptoms (maximum score of 4) with urgency and incontinence affecting quality of life. There was also a nonsignificant increase in the proportion that scored 3 for urinary symptoms from 2/21 to 14/27 patients at 2 and 7 years, respectively (Table 2). When urinary symptoms were classified by nonsignificant or significant maximum urinary symptom scores (0,1 and ≥2, resp.) there was no significant change between groups at baseline, 2-year and 7-year followup (Figure 4(a)).

LENT/SOMA median scores for bowel function were also less than 1 at baseline, 2 years and 7 years (Figure 1). However, there was a significant increase in median score from baseline to 2 years (0 to 0.2, *P* = 0.015) and baseline to 7 years (0 to 0.3, *P* < 0.001) although the increase between 2 years and 7 years was nonsignificant (*P* = 0.07). Similarly, there was a significant increase in maximum scores between baseline and 7 years (*P* < 0.01) but not between baseline and 2 years (*P* = 0.03) or 2 years and 7 years (*P* = 0.37) (Table 2). When bowel symptoms were categorised by nonsignificant or significant maximum scores (0,1 and ≥2, resp.), 6 patients (29%) had worsening in symptoms between baseline and 2 years, 4 (19%) between 2 years and 7 years and 10 (37%) between baseline and 7 years of followup, which was significant (*P* = 0.012; Figure 4(b)).

There was marked sexual dysfunction at baseline, defined as the start of radiotherapy, although all patients were prescribed neoadjuvant LHRH analogues. Significant improvement in LENT/SOMA median scores was seen between baseline and 2 years (2.67 to 2, *P* < 0.01), 2 years and 7 years (2 to 1.75, *P* < 0.01), and baseline and 7 years (*P* < 0.001) (Figure 3). The proportion of patients with a maximum score of 4 decreased with followup (Table 2), but this trend was nonsignificant (*P* = 0.44). All patients had significant sexual dysfunction with scores of ≥2 at baseline, 2-year and 7-year followup (Figure 4(c)).

## 5. Discussion

These results represent the first mature outcome and toxicity data in patients treated for localised prostate cancer with dose-escalated hypofractionated radiotherapy within a clinical trial. The use of a hypofractionated schedule is based on increasing evidence that the *α*/*β* ratio for prostate cancer is low and less than that of surrounding normal tissues, which suggests that treatment with larger and fewer fractions should be biologically advantageous [7–10]. In this cohort of 60 patients with high risk prostate cancer, we report 5-year overall survival results of 82.7% and 75.2% and bPFS 50.1% and 58.3% in patients treated with 57 Gy in 19 daily fractions and 60 Gy in 20 daily fractions, respectively. In order to compare our results with published data, it is useful to consider the total biologically effective dose in 2 Gy fractions. Assuming an *α*/*β* ratio for prostate cancer of 1.5, equivalent total doses for 57 Gy in 19 fractions and 60 Gy in 20 fractions are 73 Gy and 77 Gy, respectively.

There are five phase III randomised clinical trials comparing hypofractionated and conventional fractionation schedules [18, 26–29]; only two have reported 5-year outcome data [26, 27]. The Canadian trial reported 5-year overall survival of 88% and freedom from biochemical failure of 44% and 58% (Vancouver and Houston definitions [28, 29], resp.) in the group receiving hypofractionated radiotherapy. However, patients were treated to a low equivalent total dose of 62 Gy using a 2D technique [27]. Similarly, the Australian trial reported 5-year overall survival of 86% and freedom from biochemical or clinical failure of 57% in the hypofractionated arm (Phoenix definition). However, compliance with followup was inadequate (PSA data obtained from 96/182 surviving patients at 5 years), and patients were treated with a relatively low equivalent total dose of 67 Gy mainly using a 2D technique [26]. Long-term outcome data from nonrandomised series have also been reported [12, 14, 16]. In two series, patients were treated to a relatively low total dose (equivalent total dose of 66 Gy and 62 Gy, resp.) and 5-year actuarial bPFS in high-risk prostate cancer subgroups were 39% and 31%, respectively [12, 14]. In the third series, a subgroup of 34 patients with high risk disease was treated to 70 Gy in 28 fractions (2.5 Gy per fraction; equivalent total dose 80 Gy) and 5-year bPFS (Phoenix definition) was 75% [16].

Evidence from randomised phase III clinical trials suggests that dose-escalated radiotherapy using conventional fractionation improves local and biochemical disease control in prostate cancer [1–4]. The MD Anderson Cancer Center reported 5-year freedom from failure of 69% (extrapolated from graph) in a high-risk subgroup of 53 patients treated without hormone therapy in the dose-escalated 78 Gy arm using a 3D conformal radiotherapy boost technique [4]. A study from The Netherlands, which allowed hormone therapy (prescribed in 143/664 patients with predominantly high-risk prostate cancer and commenced up to 7 months prior to radiotherapy and continued in the adjuvant setting for either 6 months or 3 years) reported 56% freedom from failure (biochemical failure defined by ASTRO criteria) at 5-years in a high-risk subgroup of 177 patients treated within the dose-escalated 78 Gy arm using 3D conformal radiotherapy [2]. In the dose-escalated 74 Gy arm of the MRC RT01 trial, where patients received neoadjuvant hormone therapy for 3–6 months and were treated with 3D conformal radiotherapy, 5-year bPFS in a high risk subgroup of 184 patients was 57% [3]. Interestingly, in a recent update of the RT01 trial, at a median followup of 10 years, there was no improvement in overall survival in the dose-escalated arm although bPFS remained significantly superior [15]. While we note the small number of patients within this phase II clinical trial and that it was conceived before long term adjuvant hormones became standard of care [30, 31], our bPFS outcomes are comparable to published series of high-risk subgroups.

Toxicity retrospectively assessed by RTOG criteria at 7 years was low, only 9 (20%) patients reported RTOG grade 1 bladder or bowel toxicity and there was no grade 2 or higher toxicity documented. The Canadian trial, which treated 466 patients to a total equivalent dose of 62 Gy at 2.6 Gy/fraction using a 2D radiotherapy technique, reported at median followup of 5.7 years a cumulative rate of grade 3 or 4 bladder and bowel late toxicity of 1.9% and 1.3%, respectively [27]. A series of 101 patients treated to a total equivalent dose of 66 Gy at 3.13 Gy/fraction with 3D conformal radiotherapy with a median followup of 4 years, described RTOG grade 2 and 3 bladder toxicity as 9% and 1%, respectively, and RTOG grade 2 bowel toxicity 5% with no grade 3 or above toxicity [12]. In a further series of 100 patients treated to a total equivalent dose of 80 Gy at 2.5 Gy/fraction using inverse planned IMRT, 5-year RTOG combined grade 2 and 3 bladder and bowel toxicity was 8% and 5%, respectively [16]. The MRC RT01 study, where patients were treated to a total dose of 74 Gy with conventional fractionation using 3D conformal radiotherapy, reported no increase in grade 3 or 4 bladder toxicity and a cumulative increase of 4% in grade 3 bowel toxicity between 2 years and 5 years of followup [3]. Memorial Sloan-Kettering Cancer Center described in 170 patients treated to 81 Gy with conventional fractionation using inverse planned IMRT, 10-year grade 2-3 bladder and bowel toxicity in 14% and 3% of patients, respectively [19]. Our RTOG late toxicity results appear in keeping with the published literature. However, we acknowledge the limitations of these results, which include retrospective collection of data at a single time point. It is likely that there is underreporting of toxicity by the patient to the clinician and poor documentation of late side-effects within the clinical notes. The RTOG scale itself does not include certain common symptoms for example, bowel urgency or incontinence or consider sexual function. This study attempted to address these shortcomings by analysis of patient reported toxicity using a validated LENT/SOMA questionnaire [22].

In analysing LENT/SOMA data it is important to consider both median and maximum scores as these tend to under- and overrepresent toxicity, respectively [22]. For urinary function, there was no significant change in median or maximum scores between baseline, 2 years, and 7 years of followup, which suggests that bladder late toxicity was acceptable. However, there was a nonsignificant trend to increased bladder toxicity at 7 years. One strategy to further reduce bladder toxicity would be to treat with a partially-filled bladder, but this may be at the expense of inter or intrafraction variability. Median LENT/SOMA scores for bowel function remained <1 with any increase between 2 and 7 years not reaching significance and maximum scores remained stable between 2 and 7 years, suggesting that bowel late effects from radiotherapy did not increase significantly in this time for most patients. Median scores for sexual function significantly improved between baseline and 2 years and between 2 years and 7 years, probably due to discontinuation of androgen suppression. Whilst we recognise the small sample size and possibility of self-selection bias in the return of questionnaires, these results suggest that patient reported toxicity following dose-escalated radiotherapy using an IMRT technique is acceptable at 7 years and did not appreciably change between 2 and 7 years of followup. Direct comparisons between LENT/SOMA scores from published studies are difficult because study designs and patient populations are different and questionnaires are not reported in exactly the same way. Final results from the Australian trial, which assessed toxicity using a modified LENT/SOMA questionnaire at median followup of 90 months, demonstrated worsening of total bowel and urinary symptoms in both standard and hypofractionated radiotherapy treatment groups at 1 month post radiotherapy. This persisted for the follow-up period but was only significantly worse in the hypofractionated treatment group for bowel symptoms at 1 month post radiotherapy [32]. In the dose-escalated arm of the MRC RT01 trial between 2 and 5 years of followup there was an increase in the proportion of patients reporting LENT/SOMA bowel and urinary symptom maximum scores of ≥2 (45% versus 68% and 77% versus 91%, resp.) [3]. Although patient-reported toxicity amongst most patients remains within acceptable limits, there is no doubt that a small number of patients experience significant long term side effects with dose-escalated radiotherapy. Further work is required to explore factors which predict for significant late toxicity and will be work of future translational studies.

This study confirms that dose-escalated hypofractionated radiotherapy using IMRT is deliverable with comparable survival outcomes and a satisfactory level of late toxicity similar to other studies with over 5 years of followup. The radiotherapy schedules used in this early phase clinical trial are now being compared to standard treatment using conventional fractionation (74 Gy in 37 fractions) within an on-going UK multicentre phase III clinical trial [18]. Preliminary toxicity data from the first cohort of patients treated within this study suggest a similar level of toxicity using these hypofractionated regimens [18].

## Figures and Tables

**Table 1:** Baseline patient characteristics.

Disease characteristic	Number of Patients (%)
Stage	
T1	0 (0)
T2	19 (32)
T3	41 (68)

Gleason score	
2-6	18 (30)
7	27 (45)
8–10	15 (25)

Presenting PSA (*μ*g/L)	
<10	12 (20)
10–20	14 (23)
>20	34 (57)

**Table 2:** Late effects in normal tissues subjective, objective, management and analytic scales (LENT/SOMA) data: number of patients with maximum scores per symptom area (percentage of patients in parentheses).

	Bowel function			Bladder function			Sexual function		
Grade	Baseline	2 years	7 years	Baseline	2 years	7 years	Baseline	2 years	7 years
0	15 (56)	10 (48)	8 (29)	2 (7)	1 (5)	2 (7)	0 (0)	0 (0)	0 (0)
1	4 (15)	1 (5)	3 (11)	6 (22)	6 (29)	5 (19)	0 (0)	0 (0)	0 (0)
2	4 (15)	2 (10)	7 (25)	9 (33)	12 (57)	5 (19)	1 (4)	2 (11)	5 (21)
3	4 (15)	5 (24)	8 (29)	10 (37)	2 (10)	14 (52)	1 (4)	2 (11)	2 (8)
4	0 (0)	3 (14)	2 (7)	0 (0)	0 (0)	1 (4)	22 (92)	14 (78)	17 (71)
